# When sociality fails to produce solidarity: environmental NGOs and the politics of public engagement in contemporary China

**DOI:** 10.1057/s41292-025-00358-1

**Published:** 2025-06-05

**Authors:** Loretta Ieng Tak Lou, Anna Lisa Lora-Wainwright

**Affiliations:** 1 Department of Anthropology, University of Durham, Stockton Road, Durham, DH1 3LE UK; 2 School of Geography and the Environment, University of Oxford, South Parks Road, Oxford, OX1 3QY UK

**Keywords:** Environmental advocacy, Toxicity, Chemical industry, Solidarity, Environmental justice, China

## Abstract

This article examines the potentials and limitations of the concept of “chemosociality” (Shapiro and Kirksey, Cultural Anthropology 32:481–493, 2017) through a case study of Emerald, an environmental NGO addressing pollution in China’s chemical industry. Based on fieldwork and online research conducted since 2016, the article explores how chemosociality provides a lens to move beyond binary views of a homogeneous frontline community battling against toxic industries. However, it argues that chemosociality alone is insufficient to account for the unevenness of community engagement in environmental advocacy. Drawing on Bradley’s concept of biosolidarity (Bradley, Anthropology & Medicine 28:543–557, 2021), the article introduces chemosolidarity and ecosolidarity to highlight Emerald’s efforts to foster public participation. While Emerald’s attempts to cultivate chemosolidarity were unsuccessful, they nurtured ecosolidarity, grounded in a shared appreciation of nature. This example underscores the need to differentiate between sociality and solidarity and to pay attention to *affective* rather than *effective* participation to gain a more nuanced understanding of environmental justice and the complexities of toxicity and environmental advocacy.

## Introduction

Pollution in China regularly hits international headlines. Episodes of particularly intense air pollution in major urban centres, dangerous levels of contamination in the water and soil in industrialised areas and sites of extraction, and critical events (such as the deadly explosions at a chemical storage site in Tianjin in 2015), have created the image of China as a dangerous and toxic place. Over the past three decades, civil society organisations have developed a range of activities to promote environmental protection, mobilising Chinese citizens to demand cleaner and healthier environments. However, NGOs and citizens—let alone governments and other stakeholders such as the industries—do not always share the same concerns or have the same visions and agendas in tackling toxic pollution. In such cases, how do environmental organisations adapt their tactics and strategies in promoting public participation? This article addresses this question through a case study of Emerald, an NGO devoted specifically to tackling environmental pollution in China’s chemical industry.

Attention to the nuances and complexities of the work of community activists is particularly important in contemporary China, where much existing research on Environmental NGOs (ENGOs) predictably focuses on their relationship with the authoritarian state (Ho 2008; Hildebrandt 2011; Spires 2011; Hsu and Hasmath 2014; Wang and Connell 2016). The emergence of ENGOs in China has been driven by decades of environmental degradation (Geall 2013; Economy 2005; Li and Shapiro 2020). For this reason, the Chinese Party-state has shown a particular “conditional tolerance” (Lu and Steinhardt 2022) for environmental NGOs whose goals align with its own (what Peter Ho (2008) called “embedded activism”). However, over the past decade, the state has tightened legal and political control over ENGOs’ activities and financing capacities, in line with ever-tighter control on civic participation in general (Fu and Distelhorst 2017; Hsu et al. 2022; O’Brien 2023). Officials have become significantly less tolerant of independent environmental data disclosure, confrontational tactics, and social mobilisation that criticises the state (Fu and Distlehorst 2017, Li and Shapiro 2020, Wang et al. 2020). Despite this, we posit that the dominant focus on ENGOs’ relationships with the state overlooks the influence of many more factors, not least ENGOs’ resourcefulness (Wu 2013, p. 92) and alliances with other NGOs (Lu and Steinhardt, 2020), in shaping their agency. In order to fully comprehend the transformative potential of ENGOs in China, it is crucial to examine their interactions with actors and institutions beyond the authoritarian government (Wu 2013; Gaudreau and Cao 2015). In this vein, this article explores how Emerald engages with other non-governmental stakeholders, including chemical enterprises, chemical industry parks (CIPs), and local communities.

Scholarship on ENGOs in China to date has shown that they engage in a number of different forms of activism. Environmental education (including nature conservation, promoting green lifestyles, and organising community clean-up efforts) is perceived as less politically sensitive than other approaches (Wang and Connell 2016, 217) and is a common form of engagement among ENGOs in China. A relatively widespread role that Chinese ENGOs embrace is that of environmental watchdogs. However, there is considerable variation in how this role is performed. Reporting findings to the media or through public posts on social media could be dangerous because it places the NGO in a hostile relation with the polluting firms and potentially also with the local government bureaus which are responsible for environmental protection. Reporting issues directly to local environmental authorities and to the firms, by contrast, positions NGOs as collaborative and supportive of the state’s regulatory efforts—as troubleshooters rather than troublemakers. As we shall see, the latter is the stance taken by our research subject, Emerald. In the rest of the article, we illustrate how Emerald came to embrace this particular approach and examine some of the challenges it faced in translating its vision of public participation into reality. In doing so, we draw attention to the potential that its advocacy efforts hold for creating solidarity, reframing the potential harm presented by chemical industries, and shaping forms of environmental justice.

The article is structured in five main sections. Following an introduction of the conceptual frameworks and methodology, Sect. ‘An unrealised ambition: Creating an environmental impact assessment to troubleshoot for China’s chemical industry parks’ outlines the development and implementation of the Greenfield Index—an environmental impact assessment tool that exemplifies Emerald’s initial efforts to foster chemosolidarity and promote effective participation, and the challenges it faced in doing so. In Sect. ‘Chemosociality without chemosolidarity’, we further elaborate our conceptual frameworks—*chemosolidarity* (our term, inspired by Bradley’s [2021] concept of biosolidarity) and *chemosociality* (Shapiro and Kirksey 2017)—to critically examine the uneven outcomes of these participatory efforts. We argue that while chemosociality captures the complex entanglements among communities, industries, and the environment, these entanglements do not necessarily translate into sustained collective action (i.e. chemosolidarity). Sects. ‘Representing chemosociality and inviting (future) care’ and ‘Nurturing ecosolidarity through attunement to nature’ explore how the limitations of the Greenfield Index prompted Emerald to reframe its engagement strategy through two initiatives: a photo exhibition and river walks. These initiatives succeeded in fostering more affective, experiential forms of public participation—what we term *ecosolidarity*. These two vignettes are central to our conclusion that affective participation offers a meaningful and politically viable form of environmental engagement in contemporary China.

### Conceptual frameworks

Research on global environmental justice has illustrated the diversity of forms of political engagement, spanning across direct appeals to local governments and environmental authorities, environmental litigation and more contentious forms of action such as calling upon journalists to expose toxicity or engaging in street protests and other forms of direct action against polluters (for a recent and comprehensive overview see Martínez-Alier 2023). Interactions with toxicity can also be less contentious, such as when they involve environmental education and policy advocacy on the part of civil society (Yang and Calhoun 2007; Han et al. 2014), and direct engagement between affected citizens and polluters to agree compensation deals (Van Rooij et al 2012). These studies, however, tend to focus on critical events and often presuppose rational liberal subjects or publics as the agents of change. This can result in overlooking more subtle forms of engagement rooted in the “mundane, boring, everyday chores of care” (Liboiron et al. 2018, p. 333), including those that nurture mutual understanding and ethics of care for the environment—even when they may not be effective forms of environmental governance (as is the case for the initiatives promoted by Emerald).

In response, scholars have increasingly called for research on “enfeebling encounters” (Shapiro 2015, p. 369), “based in a politics not of effectiveness or change… but in obligation and ethics” (Liboiron et al. 2018, p. 340). Equally, recent studies have highlighted the diversity of social, economic, and political relations which emerge in polluted environments. Notions such as “living with pollution” (Lora-Wainwright et al. 2012), “resigned activism” (Lora-Wainwright 2021), “quiet activism” (Pottinger 2017), and “intimate activism” (Tironi 2018) all speak to individuals’ and communities’ ways of responding to environmental injustice that extend beyond conventionally defined environmental advocacy. They describe actions which may seem ineffective—such as, in Tironi’s case (2018), daily efforts to clean the leaves of tomato plants which will soon be dusty once again—but which are driven by ethical obligations towards human and non-human others rather than striving for effective action. Conversely, Lou suggests that those living in contaminated surroundings in China actively decide to ignore (“contrived ignorance”) or “unnotice” (Lou 2022) pollution as a strategy for coping with toxic exposures (see also Auyero and Swistun 2009; Tironi and Rodríguez-Giralt 2017; Valdivia-Rivera et al. 2018).

In some cases, environmental advocacy is rooted in “biosociality” (Rabinow 1996): social relations premised upon a shared biological condition and the scientific discourses and technological infrastructures through which it is understood and managed. In her study of post-Soviet Ukraine, Petryna (2002) examined how biosociality was mobilised by Chernobyl victims to access state compensation as demands for “biological citizenship”. Rose and Novas expanded the concept to describe “novel practices of biological choice” which involve “a prudent and yet enterprising individual, actively shaping his or her life course” (2005, p. 458), as well as groups of people who come together to advocate for their rights on the basis of a shared condition or diagnosis. In the same spirit, the concept of “chemosociality” draws attention to “the longstanding relationships and emergent social forms that arise from chemical exposures and dependencies” (Shapiro and Kirksey 2017, p. 484; see also Ballayanis and Garnett 2020).

This article applies the concept of chemosociality to make sense of the pathways of advocacy adopted by the Chinese environmental NGO, Emerald. We show that the concept is useful for illustrating Emerald’s efforts to take on board the entanglements between local communities, chemical industries and local environmental governance, but ultimately it is not sufficient for making sense of the limited public participation in some of Emerald’s initiatives. We argue that it is necessary to disentangle sociality defined as co-presence from solidarity defined as active efforts in sustaining and enacting particular visions of the environment.

Inspired by Bradley’s work on “biosolidarity” (2021), we distinguish two types of solidarity: chemosolidarity (which takes chemicals as starting points for participation) and ecosolidarity (which designates a shared appreciation of nature as the basis for solidarity).^1^ These concepts help us to illustrate the diverging outcomes of Emerald’s advocacy to nurture public participation. They allow us to unpack diverse and shifting relationships between environmental NGOs, industries, local governments and communities and to understand why activism may take on particular guises. In turn, we suggest that by distinguishing between sociality and solidarity, we can better understand the entanglements between communities, activists and toxicity, without assuming that communities want to focus on environmental damage and without focusing on civil society’s relative *effectiveness* in environmental governance. In this way, attention to the formation of chemosolidarity and ecosolidarity (or lack thereof) can enable a broader understanding of environmental justice, offering new insights into the complexities of toxicity and environmental advocacy.

By critically examining the formation and limitations of chemosolidarity and ecosolidarity, this article underscores the importance of reflecting on the conditions under which solidarity emerges or fails to materialise. Understanding these dynamics not only deepens our comprehension of the relational and affective aspects of environmental activism but also informs strategies for more attuned and inclusive advocacy that aligns with the diverse needs and priorities of affected communities.

### Methods, fieldsites, and ethical considerations

This article draws on roughly 2.5 months of on-site fieldwork, a dozen of in-person interviews conducted in an eastern coastal Chinese city in March and October 2016 and in November 2018, and several online follow-up interviews in June 2020 due to the COVID-19 Pandemic. Lou was introduced to Emerald by Dr. Xinhong Wang, a former Research Fellow for the ERC funded project Toxic Expertise, who conducted research with this environmental NGO in March and October 2016. We thank Xinhong Wang for laying the foundation for Lou’s subsequent fieldwork. The research was reviewed and approved by Lou’s former employer, the University of Warwick, and its ethics committee. Our interlocutors were mostly key workers of Emerald and residents in the area, but this article draws mainly from interviews with the NGO staff. All interviews were carried out in Mandarin. We sought to compensate for the limitations of “helicopter fieldwork” with in-depth semi-structured interviews with NGO workers (interviews lasted between 2 and 3.5 h), extensive reviews and content analysis of the NGO’s publications, promotional materials, and original content on its WeChat Public Account (containing 336 original postings) between 2020 and 2022. The time span of the research enables us to trace the trajectory of Emerald’s endeavour to tackle chemical industry park pollution and form chemosociality, chemosolidarity and ecosolidarity. During the analysis and writing-up stage, both authors analysed some of the WeChat Public content together and refined the arguments and analytical framework collaboratively, drawing insights from their own research on pollution and environmental activism in China and Hong Kong over the past decade.

Given the sensitivity surrounding civil society in China, it is essential for us to consider the potential effects of our scholarship on the civil society actors we focus on. The implementation of the 2017 Foreign NGO Law raised concerns among NGO workers regarding communication and information sharing with foreign researchers. In view of this, all names of individuals and organisations have been pseudonymised, except for the environmental NGOs that were already discussed in other publications. Some details about Emerald, original quotations from their publications and exact dates on WeChat posts have been withheld in order to preserve anonymity. However, it is important to acknowledge that achieving true anonymity may be challenging in a country with pervasive monitoring and surveillance systems. Given that the NGO itself—as this article illustrates—is particularly careful in positioning itself as a partner to the state and industry, and a supporter of sustainable development, the political sensitivity of their work is relatively low. Nevertheless, we have taken great care to ensure that the content and arguments presented in this article do not have any negative repercussions for the individuals and organisations discussed (The China NGO project 2017).

## An unrealised ambition: creating an environmental impact assessment to troubleshoot for China’s chemical industry parks

Our research subject, Emerald, is a registered grassroots environmental organisation dedicated to addressing local environmental issues. Its core work focuses on preventing industrial pollution by monitoring the environmental performance of industrial enterprises. According to its official website, “it promotes environmental remediation in polluting companies through a mixed method approach, including on-site investigations, data analysis, reporting, environmental audits, environmental management training, roundtable discussions, and public participation” (original quote on file with the authors).

Founded in 2000, Emerald originally focused on environmental education, as was typical for the first generation of ENGOs in China (Geall 2013). The organisation faced a critical juncture when their overseas funding ceased in 2009, motivating them to transform into an environmental watchdog organisation. Qishan, a veteran staff at Emerald, recalled:

As an environmental organisation, we found ourselves at a crossroad. We had two options: either carry on with our education program without any funding, which would be hard if not impossible, or switch gears and venture into a new territory. After some heated debates, we decided to focus on industrial pollution, right here in our province. Environmental education was a common pursuit among environmental NGOs in China. Even if we dropped it, someone else would do it. So we asked ourselves: what unique contributions can we bring to the table? And the answer was evident: We needed to confront the local environmental issues head-on, right in our own backyard. This is our home. If we don’t step up, nobody else will (interview with Lou, 2018).

Emerald’s decision to specialise in chemical industry park pollution was a logical one given its locality. Situated in a second-tier city surrounded by chemical plants, the region has a longstanding history of chemical pollution due to its early establishment as a hub for China’s chemical industry. Over time, these chemical clusters, originally positioned away from densely populated areas, became sources of environmental and safety hazards as urbanisation encroached. The geography of this urban and peri-urban area—mostly surrounded by mountains—also hinders the dispersion of pollutants and contributed to persistent smog. In 2011, a local resident contacted Emerald, expressing her concern about a new chemical plant and increased cancer incidence among the surrounding residents. In the light of these local circumstances, Emerald recognised the urgency of tackling industrial pollution for this region.

The transition from an education-oriented ENGO to an issue-oriented ENGO was also propelled by Emerald’s collaboration with the Institute of Public and Environmental Affairs (IPE) during a funding shortage between 2009 and 2011. IPE is the most prominent and longstanding ENGO dedicated to promoting data transparency in the fight against pollution in China. Through the collection and analysis of government and corporate environmental records, IPE develops pollution databases that are accessible not only to the government but also to the public (Tarantino and Zimmermann 2017). One of IPE’s transparency initiatives, the Corporate Information Transparency Index (CITI), has been instrumental in helping the Chinese government monitor international companies. Since 2017, the CITI index has driven over 3000 factories in China to enhance their environmental performance (Ma 2017), highlighting the potential for NGOs to serve as a mechanism to encourage (if not ensure) environmental self-governance and Corporate Social Responsibility. Emerald collaborated with IPE to investigate the supply chains of Apple and exposed its environmental violations. This resulted in significant improvements on Apple’s part, earning it a top ranking in the CITI index.

Emerald’s involvement with IPE had a profound influence on its own approach to chemical pollution, as Qishan explained:

Our experience with IPE has equipped us with the vision and professional capacity to carry out our own work in the chemical industry parks. We went to the field with IPE and observed how they carried out fieldwork. It was a process of learning by doing (Interview with Lou, 2018).

Inspired by the success of IPE’s environmental performance index, Emerald designed its own framework in 2018, which we refer to as the Greenfield Index. While formally presented as an environmental impact assessment tool, the Greenfield Index functions not only as a ranking mechanism but also an organisational platform through which Emerald promotes more transparent, participatory, and accountable environmental governance in chemical industry parks (CIPs). Serving as the foundation for Emerald﻿’s intervention in the operations of chemical industry parks (CIPs), the Index provides both evaluative criteria and a collaborative framework for dialogue and reform. In formulating the Greenfield Index, Emerald drew on the Analytic Hierarchy Process (AHP), a multicriteria decision-making approach, and the input from 23 experts representing various fields, including environmental consultants, scholars, lawyers, ENGO workers, government officials, and industry representatives.

**Graph 1:**
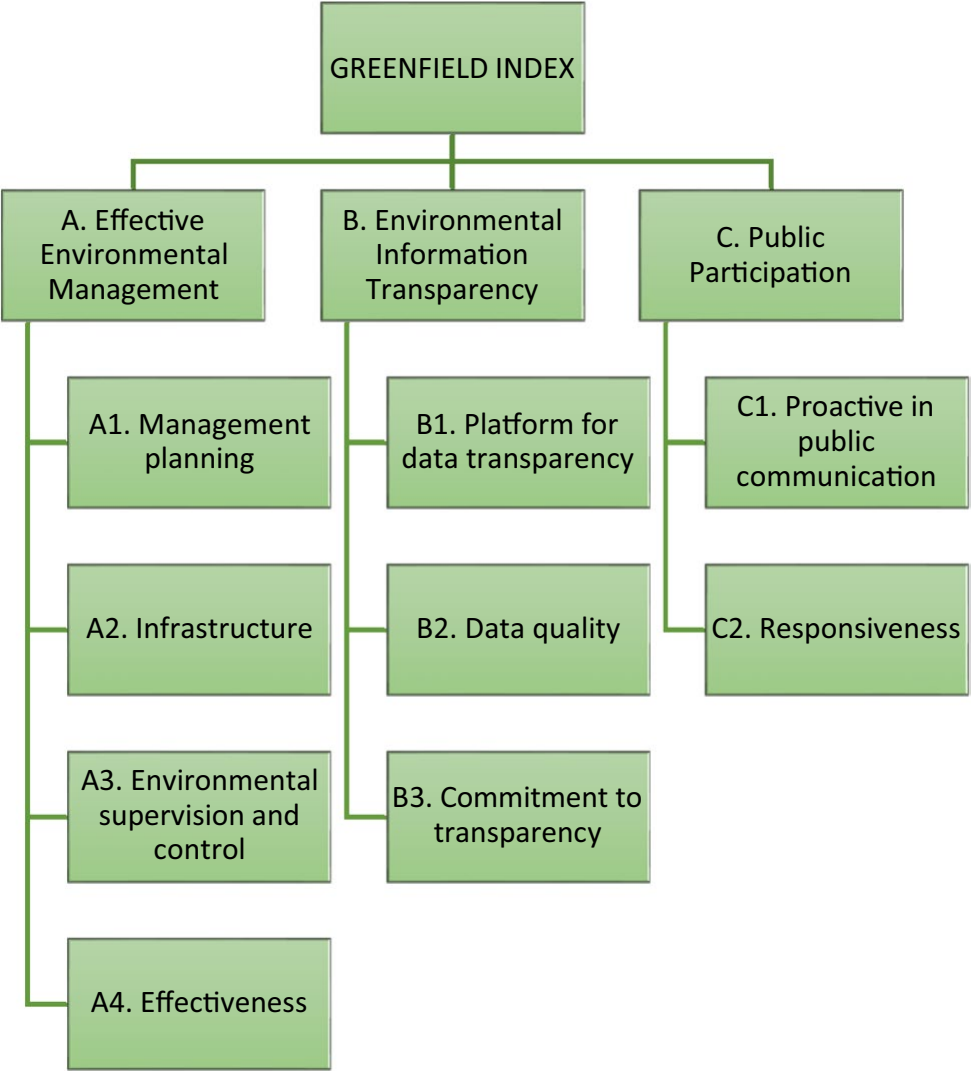
This is a simplified version of the Greenfield Index, which consists of three main categories (A, B, C). In an order of importance, indicators that weighted the most are marked as A1, B1, or C1 in their corresponding categories, followed by A2, B2, C2, and so on Compared to the provincial government’s assessment of CIPs, the Greenfield Index adopts a less technical approach and incorporates two critical criteria absent from the government's evaluation: information transparency and public participation. Information transparency is evaluated based on the presence of both online and offline platforms for disclosing environmental data, including reports, irregularities, and fines. In addition, Emerald encourages CIPs to employ members of the public as environmental protection supervisors, establish channels for public complaints, and ensure prompt responses to reported pollution. For example, air and water pollution should be investigated on-site within two hours, while other types of complaints should be addressed within two days. Moreover, CIPs are urged to conduct regular surveys to gauge community satisfaction with their performance. Qishan highlighted the importance of respectful communication during these surveys:

We make it clear that it’s not enough to just reach out to people. If their attitudes [towards NGO staff or towards the public] during the survey are poor or condescending, they will receive a lower rating on public participation. The idea is to push chemical parks and chemical enterprises to become more proactive in their communication with the general public, doing so in a polite and friendly manner (interview with Lou, 2020).

By emphasising information transparency and public participation, the Greenfield Index seeks to create a framework where chemical enterprises and CIPs are held accountable by the public. At the same time, Emerald’s approach is sensitive to the symbiotic relationship between industry, local government and local residents. Rather than taking a confrontational stance towards CIPs, Emerald acts as a troubleshooter, which aims to bridge the gap between the government, CIPs, and the local community and to provide constructive, actionable solutions. To illustrate this vision, Qishan recalled Emerald’s first ever field trip in an interview with Lou. Staff spent thirteen days collecting evidence of pollution near a CIP but held off reporting the offences to the government until they figured out a solution for the chemical industry park.

We tried to stay calm and not get too worked up when we saw the pollution. We delayed reporting the violations because we didn’t want to just reveal the problems. We wanted to solve the problems! In the end, we submitted a report to the Environmental Protection Department [at the local government] with our recommendations on how to address the issues (interview with Lou, 2020).

Years of observation led Emerald staff to realise that reporting isolated violations seldom leads to sustained improvements in the overall performance of a chemical industry park. Even if fines are imposed, the positive impact tends to be short-lived. Therefore, Emerald insists that sustainable change must come from within the park management committees, through more transparent and participatory monitoring mechanisms and improved park management. One mechanism for enabling such participatory monitoring was the proposal to appoint local residents as environmental protection supervisors. However, this proposal remained largely aspirational. The initiative failed to take root in practice, as few residents expressed interest in assuming the role—possibly due to fear of retaliation from the industry bosses. Needless to say, CIPs had little incentive to support or institutionalise the initiative. Without buy-in from either side, Emerald’s vision of public participation through the Greenfield Index proved difficult to implement. In this sense, the Greenfield Index stands as an unrealised ambition: a promising framework whose transformative potential remains constrained by uneven stakeholder engagement.

## Chemosociality without chemosolidarity

The concept of chemosociality (Shapiro and Kirksey 2017; Kirksey 2020) is useful for understanding the position adopted by Emerald and its strategies for environmental advocacy. Emerging from a multispecies approach to chemicals which invites attention to a broad set of entanglements, chemosociality helps to overcome a generic idea of “affected communities” and a dualistic approach which presupposes hostility between the “public” and polluters. As such, it aptly encapsulates Emerald’s approach: situating itself within the complex chemosocial entanglements between CIPs, local governments and local communities, as a partner to the industry and an enabler for community participation. Evidence of this approach can be found in many of their online newsletters and original posts on WeChat, in which Emerald staff described a “beautiful” and “liveable” chemosocial future with the implementation of the Greenfield Index and other initiatives:

For most people, the chemical industry is synonymous with ‘pollution,’ ‘danger,’ and ‘risking life for money.’ There have indeed been issues over the past few decades, and these are problems that both the nation and the industry are committed to changing. However, considering that the chemical industry is an integral part of our daily lives, can we envision and build a green, safe, and sustainable future with it? […] In our vision, the future chemical industry town will be liveable and economically prosperous …and the interactions and communication between the industrial park and the residents are two-way, timely, and friendly. The presence of the industrial park has become a bright and beautiful hallmark of the area (WeChat public content 2023).

Although Emerald does not use the term chemosociality in any of its publications, its vision and mission can be understood as an effort to foster chemosociality. But as we have discussed in the previous section, the Greenfield Index failed to activate these chemosocial relations. To understand this mismatch between chemosocial entanglements and the absence of active participation, we need a more precise definition of chemosociality than its original loose characterisation, which includes both “relationships” and “emergent social forms” rooted in “chemical exposures and dependencies” (Shapiro and Kirksey 2017, p. 484). Indeed, while chemosociality portrays entanglements between chemicals, humans, and the environment, it is a less precise conceptual tool for understanding why some forms of sociality do not coalesce into action.

In this article, we use the term chemosociality to refer to relationships rooted in shared experiences of chemical exposure, and we introduce “chemosolidarity” to describe the forms of action that materialise the ethical obligations sustained by chemosociality. Chemosolidarity is inspired by Bradley’s concept of “biosolidarity”. Based on her ethnographic work on hair pulling and skin picking, Bradley introduces the term “as a framework to make sense of the relationship between the body, sociality and advocacy” (2021, p. 544) and to encapsulate “the activist efforts of biosocial groups, and simultaneously recognise[s] the reproductive power this work can have on biosocial networks” (ibid.: 545).

Whereas for Bradley, “bio” draws attention to the power of biomedical labels to create solidarity as well as to the importance of the body asserting its agency. Chemosolidarity, for us, foregrounds the centrality of chemicals as foci for activists’ engagement—particularly in efforts to encourage participation in pollution monitoring among local communities and industries.^2^

Most importantly, Bradley notes that solidarity is “fragile, fraught, and is dependent on labour for its ongoing sustainability” (2021, p. 551). This emphasis on the active labour required to produce and sustain solidarity is crucial for understanding why chemosolidarity did not materialise in Emerald’s case: the labour of active participation, as envisioned by Emerald, was lacking among community members and CIPs alike. Conversely, chemosolidarity provides a valuable conceptual tool for grasping the contingent nature of solidarity, as well as the emotional and ethical dimensions of active participation, which are often absent from analyses centred on chemosociality. Kirksey himself acknowledged that chemosociality can manifest in a variety of ways: ““some social groups have coalesced around place-based political action, while other chemosocial associations have proved to be ephemeral, evanescent, and conditional” (2020, p. 24). However, the concept does not enable an analysis of this diversity of outcomes as we wish to pursue through chemosolidarity.

In our case study, chemosolidarity failed to materialise partly because of a lack of commitment by industries. Emerald has been able to establish collaborative relations with some CIPs, which willingly allow Emerald staff to undertake environmental assessments for them, exerting a form of professionalised supervision (see also Zhong and Wang 2023). However, only a dozen of chemical industry parks have welcomed Emerald’s on-site research, and even fewer have embraced their advice. Without effective incentive and penalty mechanisms, their compliance remains minimal. While IPE’s CITI Index has been effective in leveraging customers’ growing environmental consciousness to hold global brands such as Apple Inc. and Nike accountable, Chinese companies have been less responsive to such pressure. In a country where disclosure on environmental pollution has not been the norm (Larson 2010), obtaining information from the government and the private sector remains a challenging task. Wang et al. (2021) revealed that, in Jiangsu province, only 25% of CIPs “have set up specific channels for the release of environmental information” (Wang et al. 2021, p. 3). Government officials often cite reasons like national security, public security, economic security, or social stability to avoid disclosure, and many chemical enterprises and chemical industry parks are unaware of the 2015 Law for the Disclosure of Environmental Information (Meng 2011). Some companies even go as far as considering details of pollution treatment equipment and pollutant release as commercial secrets, hindering information disclosure.

The Greenfield index also fell short of producing chemosolidarity due to the limited involvement of local communities. We identified two clusters of interconnected reasons for the lack of public engagement, which aligned with previous research on local communities’ attitudes and behaviours towards chemical industry parks (He et al. 2018). The first cluster is to do with technical aspects. In a post that celebrated their organisation’s 20th year anniversary, Emerald admitted that as they deepened their focus on chemical pollution and CIPs, the distance between them and the public was growing greater (WeChat public content 2020). Staff later reflected that the public sees chemical industry parks as “a highly technical and boring topic” (WeChat public content 2023; also see Hartings and Fahy 2011). Our findings strongly echo with previous research that residents who lived in proximity to chemical industry parks in China “had little specific knowledge about the park” and relied mainly on their bodies and everyday experience to gauge how polluted the CIPs were (Wang et al. 2021; Lou 2022). As these previous studies on China have shown, chemistry or chemicals are perceived by local residents and industry workers to be beyond their understanding or remit—something better left to experts. Even for those who are willing to engage, “it is difficult for the public to obtain environmental information about CIPs” (Wang et al. 2021, p. 3), and the information obtained is generally not easy-to-understand for lay people without prior training. While the lack of information is related to the lack of disclosure noted above, the highly professionalised nature of the Greenfield Index (and environmental impact assessment more widely, as noted by Zhong and Wang 2023) exacerbated the sense that local residents could not contribute fruitfully to the Greenfield Index, undermining potential for chemosolidarity to emerge.

Beyond these technical aspects, disaggregating the spatial, social and economic positions of the public sheds some light on the reasons why local communities did not participate as Emerald had hoped to enact a shared vision of care for the environment as described by the term chemosolidarity. On the one hand, as chemical industries became increasingly concentrated in industrial parks and relatively removed from residential areas, some local communities no longer occupy a frontline position in relation to polluting firms and have therefore become less actively engaged in pollution monitoring and supervision (see Zhong and Wang 2023, p. 43). In the case of these communities, chemosociality itself is weakened, undermining the premise on which chemosolidarity may be built. On the other hand, communities which remained in close proximity with industry were unwilling to engage with the Greenfield Index as they feared what impact such monitoring might have on the industry and on their jobs (Fieldnotes 2016 & 2018, also see Wang et al. 2021; Mah and Wang 2019). As is noted elsewhere, in counties where industries have given a boost to the local economy and lifted farmers out of poverty, there is often a culture of silence and resignation regarding environmental issues (Lora-Wainwright 2021; Lou 2022). The economic benefits provided by the industry lead many residents to either accept chemical pollution as an inevitable consequence of living near an industrial park, demand economic compensation for pollution or plan to leave the area as soon as they have earned enough money (Lora-Wainwright et al. 2012; Lou 2023). In these cases, GDP growth takes precedence over environmental concerns, with villagers and county leaders expressing their preference for “dying of poison than of starvation” (quote from a printed brochure of Emerald 2018). This phenomenon is extremely common in industrial areas and is observed in both authors’ work elsewhere in China (see citations earlier in this paragraph). As a whole, these elements hindered the Greenfield index’s potential to transform chemosociality into chemosolidarity—that is, to convert shared chemical exposure into active engagement and care through pollution monitoring. In the next section, we explore Emerald’s response to this “failure”, their attempts to re-engage the public, and the emergence of a different kind of solidarity.

## Representing chemosociality and inviting (future) care

In line with its commitment to connecting the industry and the public, Emerald commissioned a photographer in June 2020 to document everyday life inside one of the local CIPs. The goal was to make this often opaque and distant industry more visible and relatable to the people whose lives are closely affected by it, yet who know little about how it operates. The photos were exhibited in a city bookshop and later shared on Emerald’s social media platforms and website. The images were accompanied by life histories and snapshots of the daily lives of local residents—from factory workers and environmental monitors to those involved in the local service sector, such as restauranteurs, as well as farmers and fishermen (WeChat public content 2020). We do not seek to determine whether this initiative succeeded in creating chemosolidarity or in nurturing better understanding and care towards residents near CIPs. This is not our aim. Rather, we find it more instructive to reflect on Emerald’s decision to organise the exhibition in the first place, and on how it represented the relationship between industries, humans and the natural environment—what we defined earlier as chemosociality.

Unlike the sensational images that often depict pollution and human misery in China, the photo exhibition offered a more grounded portrayal of life around the CIPs. Indeed, in the online post introducing the images, the photographer noted that he was unsure about what to photograph, as nothing he saw seemed to represent the chemical industry as he had imagined it. Similarly, in a diary entry describing their visit to a CIP, Emerald volunteers expressed awe at seeing an egret living amidst the industrial environment and an oleander bush in full bloom with surrounding vegetation (WeChat public content 2023). “The industrial park is not as poorly maintained as one might imagine”, a university student and Emerald volunteer wrote in their fieldnote (WeChat public content 2023). “On the contrary, the park’s greenery is quite well-maintained, and on clear afternoons, you can even hear birds chirping in the quiet park. The spacious and well-planned roads give everything a tidy appearance, and environmental monitoring facilities set up throughout the park are operating in an orderly manner. Of course, while walking in the park, there are still occasional unpleasant odours when the wind picks up. This seems *inevitable* for now, but *hopefully*, it will not be an issue in the future” (WeChat public content, 2023; our emphasis).

Notably, this photo exhibition and the accompanying stories do not illustrate the presence of chemicals or their effects, but rather their impeccable management and therefore the innocuousness of the surrounding chemicals. Indeed, one of the featured testimonies is from an environmental protection officer, who is portrayed dutifully patrolling CIP premises and sharing his record-keeping with his superiors during a routine investigation. The images show young, professional, uniformed staff wearing safety gear while examining orderly documents and pristine operation rooms—scenes that would not look out of place in promotional material produced by the CIP or in official propaganda material produced by the Chinese party-state. Through conversations with park managers, some volunteers also learned that in recent years, the park has been focusing on the topic of responsible care. As a whole, the exhibition and its accompanying narratives promote a vision of progress and peaceful coexistence between communities, the natural environment, and the industry. Contrary to romaniticised notions of nature as separate from industrial development, the images depict wild fauna and blossoming flowers integrated into the chemical industry’s infrastructure. While acknowledging that some “unpleasant odours” are currently “inevitable”, the exhibition expresses hope that these, too, will eventually be resolved.

As with any form of representation, the photos do more than *portraying* the existing chemosocial entanglements between the industry, humans, animals, and the natural environment. They simultaneously *construct* and *produce* a particular vision of what these relationships are and what they could or should become. On the one hand, we argue, Emerald promotes these images as evidence that the industry *can* and *does* coexist with people and nature. On the other hand, they serve as a form of prefigurative engagement with the industry as a responsible neighbour, aiming to hold it accountable for maintaining such harmonious relationships into the future. This aligns with the Chinese state’s vision of sustainable development and ecological civilisation. The relations of care and politics of hope that it seeks to nurture centre on the possibility of harmonious chemosocial coexistence, a scenario in which natural beauty can be appreciated while the benefits of the chemical industry are pursued.

## Nurturing ecosolidarity through attunement to nature

Emerald have been committed to public participation since their foundation. As a member explained during an interview, “China’s environmental governance cannot be solely top-down. It needs public participation” (Interview with Lou, 2020). They reiterated this commitment in their online posts: “When an environmental organisation lacks public support, its advocacy efforts are severely weakened” (WeChat public content 2023). For this reason, Emerald decided to relaunch their local environmental education programmes after repeated failures to engage the public in their work on CIPs and the Greenfield Index. Unlike the earlier, top-down models of environmental education, the current approach is more oriented towards an equal and open dialogue with the public.

Online accounts of one of Emerald’s open days reflect the open and consultative relationship it aims to foster with the public. Photos shared on its WeChat account show over 20 participants of varying genders and age, with women outnumbering men; most attendees were in their 20 s and 30 s. The event featured playful but engaging educational activities designed to encourage open dialogue, the sharing of affective experiences, and collaborative decision-making. Participants watched a short film about Emerald’s past and current initiatives and placed post-it notes under photos of previous activities to indicate which one they would be most interested in joining. Among the activities presented, river walks and plastic reduction initiatives were most popular.

Comparing to the earlier model of environmental education, which relied on top-down information delivery, Emerald’s current approach is far more horizontal, even bottom-up. It aims to respond to community interests and foster a sense of solidarity. Since river walks and plastic reduction were initiatives that attracted substantially more public engagement (WeChat public content 2019, 2020, 2021, 2023), they were the first local environmental education programmes that Emerald resumed in recent years. The plastic reduction initiatives invited people to reflect on the life cycle of plastics, their environmental impacts, and ways to reduce the use of single use plastic—particularly in the milk and fruit tea industry. The river walks initiative offered guided walks for community members and school groups twice a month, during which participants are invited to closely observe the local vegetation and its habitat, test river water samples, and learn how to interpret the results. While our data on the former are limited, we have some direct experience of the latter initiatives, which will therefore be our focus.

“River Walks” are a relatively widespread activity for Chinese NGOs (see Fossati 2018; Von Mangoldt 2016), but the emphasis and atmosphere on such walks can vary depending on the guides and the participants. Emerald’s river walks showcased participants smiling, listening intently to their guides’ introductions to the local ecology. The guides themselves often use “nature names” like Sunflower rather than their own given names or surnames, a practice observed among environmental advocates in Hong Kong (see Lou 2016). The naming practice signals a sense of closeness to nature while also offering a degree of anonymity for the advocates. These walks along waterways and old walls in the city, along with family-oriented educational sessions on composting and making eco-enzyme (Lou 2016), produced “ecosolidarity”: they nurtured awareness among nearby city residents of both the often-unnoticed beauty of nature and the presence of contamination. Where chemosolidarity leverages the centrality of chemicals as focal point for activist engagement in pollution monitoring, ecosolidarity designates a feeling of oneness between humans and their natural surroundings, and among humans united by a shared desire to protect nature. It materialises through shared awe for nature and practical actions taken to care for it. Evidence of ecosolidarity may be found in the popularity of river walks, as reflected in the recollections posted online by volunteers and participants. Enthusiastic comments shared on the NGO’s WeChat account highlighted the beauty of their natural surroundings and expressed appreciation for the diversity of plants that can thrive even in a city environment. Participants also noted that they learned about ways to engage in ongoing acts of care for nature, such as making compost and reducing the use of plastics.

River walks conducted by Emerald also included water tests. This may be categorised as a form of citizen science because it involves the collection and testing of samples by residents (see Goron, Lora-Wainwright, and Huang 2024). However, Emerald avoids dwelling on the relative scientific merit of the data collected; water tests are described as a “fun experiment” which often involves children and the results are swiftly accompanied by cautionary statements that the data are for reference only and are liable to errors (WeChat public content 2019, 2020, 2021). On the surface, this way of presenting water testing as amateur and liable to inaccuracies is at odds with the technocratic attitudes prevalent in public debates and policymaking (Brombal 2020, p. 39). It is also in stark contrast to water testing advocated by other civil society organisations in China, which present test results as the basis upon which to demand redress from the relevant authorities (Hsu et al. 2020). Indeed, it directly undermines the potential for using such data to demand redress for pollution.

Presenting water testing as a “fun experiment” couches it within a politically safe framework, congruent with Emerald’s collaborative approach towards industries and the local state alike. But this does not prevent participants from gaining awareness of potential contamination nor from building relationships with other participants based on this shared awareness. As an Emerald staff member wrote in a published brochure, “We told the residents that they are entitled to speak about what happens at their doorsteps”. These “experiments”, in other words, can still invite reflection on water quality and foster a learning process about how water may be tested, about the parameters, metrics and measurement methods employed in data collection, and about the severe consequence of water pollution. Crucially, we argue, just as the Greenfield Index was intended less as a technocratic tool and more as a channel for interfacing between industry and local residents, water testing is less about the results per se and more about its performative, pedagogical, and moral dimensions. Indeed, we argue, the ethical and affective entanglements embodied through water testing and the walks of which it is part are more important than the potential scientific value or validity of the test results (see also Brombal 2020). These entanglements undergird ecosolidarity—they raise residents’ awareness of water pollution and instil in them a sense of belonging and emotional attachment to the natural features of their local environment. These emerging forms of ecosolidarity in turn hold potential to foster more inclusive participation, but also empower community residents to monitor and protect their living environment (see also Lora-Wainwright et al. 2021). Ultimately, and crucially, such activities can serve to both appreciate nature and address pollution. In fact, the premise of Emerald’s approach is that these two foci are interlinked because, in appreciating nature, citizens are reminded of the threat of pollution. As testament to this, some river walks volunteers later became core volunteers for Emerald’s CIP project.

## Conclusion: from effective to affective participation: reframing environmental justice activism

The experience and strategies adopted by Emerald offer valuable food for thought on how we might reframe approaches to pollution and environmental justice activism. Initially, Emerald focused on the Greenfield index, encouraging industries to be transparent about their emissions and inviting citizens to participate in holding them accountable. However, this initiative resonated little with residents living near chemical industry parks. When efforts to engage the community in this way failed to bear fruit, Emerald began experimenting with alternative forms of participation and environmental awareness, namely through photographs and stories of the CIP residents and workers, plastic reduction initiatives and river walks. The photo exhibition depicted and promoted the possibility of harmonious coexistence between industry and natural beauty, while river walks nurtured nature-based ecosolidarity, giving voice to emotional and affective attachments, as evidenced in participants’ online posts extolling the beauty and diversity of the natural environment.

Emerald’s initiatives reflect a shift from engaging communities and industries in *effective* environmental governance to fostering *affective* engagement with chemosocial entanglements, the livelihoods supported by industry, and an ethical commitment to care for nature. While its attempts to address chemical pollution may not be effective in the conventional sense of curbing emissions or enforcing accountability, they cultivate a particular ethics of care and solidarity. Although these efforts would likely not match standard definitions of environmental justice activism (e.g. Alier 2023), nor can the individuals and communities interpellated by Emerald be understood as the rational neoliberal subjects imagined in conventional forms of political participation, these subtle forms of engagement articulate what Tironi (2018) memorably termed “hypo-interventions”.

Shifting attention from measuring the effectiveness of public participation in environmental governance to nurturing ethical and affective attachments engages in a different kind of politics: the politics of hope (Lou 2016). However, ephemeral and elusive, such attachments can have profound repercussions in the longer term as they shape visions of and hopes for the future and one’s place in it. For these reasons, we argue these subtle forms of engagement deserve more attention than they currently receive: they showcase the importance of building solidarity implicitly and responsively, in relation to community interests and responses. In authoritarian settings such as contemporary China, where conventional forms of resistance and collective action are largely beyond reach, these seemingly ephemeral and yet powerful affective entanglements with nature and more-than-human others take on additional significance, as incipient seeds for different visions of the future.

Challenging technocratic and scientific hegemony which dictates attention to objective measures of toxicity, Shapiro et al. (2017) wrote about the value of “inviting apprehension” rather than focusing on technical knowledge to establish evidence of contamination. As our case study has shown, Emerald’s evolving strategy and determination to encourage public participation demonstrate the tremendous value of inviting individuals and communities to explore, admire, and develop emotional attachments to their natural surroundings. This is valuable not only because such approaches may ultimately be a productive means of stimulating public participation, but also because they offer the most politically safe way for civil society actors to operate in contemporary China today. By aligning itself with the state’s commitment to building an “ecological civilisation” and supporting industrial development and by championing *affective* rather than *effective* participation, Emerald is able to nurture *ecosolidarity* without casting local communities as victims of pollution and or provoking state repression.

Beyond its empirical utility to this case and to authoritarian contexts, our conceptual framework makes an important theoretical contribution to studying activism and to understanding when and why they may not emerge in a form that would be recognized as part of a liberally-defined civil society. Differentiating chemosociality from chemosolidarity and ecosolidarity—and thereby distinguishing relationships born of co-presence from emergent practices of support and care—allows us to better account for scenarios where co-presence does not lead to active engagement. At the same time, this distinction allows us to attend to affective, quiet, intimate and relational forms of participation. Such an approach enables researchers to reflect on the assumption that sociality is a prerequisite for solidarity and, more importantly, to critically examine and challenge dominant definitions of fenceline communities and environmental justice activism.

## Footnotes

1 We recognise that these forms of solidarity are not always distinct, but it is useful for our analytical purposes and given our empirical data to distinguish between them.

2 Chemosolidarity could also include activism related to beneficial chemicals, such as the positive impacts of fluoride in water, though this form of chemosolidarity is not relevant in our case study.
